# High-glucose diets induce mitochondrial dysfunction in *Caenorhabditis elegans*

**DOI:** 10.1371/journal.pone.0226652

**Published:** 2019-12-17

**Authors:** Jonathan Alcántar-Fernández, Angélica González-Maciel, Rafael Reynoso-Robles, Martha Elva Pérez Andrade, Alain de J. Hernández-Vázquez, Antonio Velázquez-Arellano, Juan Miranda-Ríos

**Affiliations:** 1 Programa de Doctorado en Ciencias Biomédicas, Universidad Nacional Autónoma de México (UNAM), Ciudad de México, México; 2 Unidad de Genética de la Nutrición, Depto. de Biología Molecular y Biotecnología, Instituto de Investigaciones Biomédicas, UNAM e Instituto Nacional de Pediatría, Ciudad de México, México; 3 Laboratorio de Morfología Celular y Tisular, Instituto Nacional de Pediatría, Ciudad de México, México; Tokyo University of Agriculture, JAPAN

## Abstract

Glucose is an important nutrient that dictates the development, fertility and lifespan of all organisms. In humans, a deficit in its homeostatic control might lead to hyperglucemia and the development of obesity and type 2 diabetes, which show a decreased ability to respond to and metabolize glucose. Previously, we have reported that high-glucose diets (HGD) induce alterations in triglyceride content, body size, progeny, and the mRNA accumulation of key regulators of carbohydrate and lipid metabolism, and longevity in *Caenorhabditis elegans* (PLoS ONE 13(7): e0199888). Herein, we show that increasing amounts of glucose in the diet induce the swelling of both mitochondria in germ and muscle cells. Additionally, HGD alter the enzymatic activities of the different respiratory complexes in an intricate pattern. Finally, we observed a downregulation of ceramide synthases (*hyl-1* and *hyl-2*) and antioxidant genes (*gcs-1* and *gst-4*), while mitophagy genes (*pink-1* and *dct-1*) were upregulated, probably as part of a mitohormetic mechanism in response to glucose toxicity.

## Introduction

Mitochondria are essential eukaryotic organelles that participate not only in the production of energy (by means of the tricarboxylic acid cycle, beta-oxidation, the electron transport chain (ETC)), but also in diverse cellular events such as Ca^2+^ and redox homeostasis, apoptosis, the biosynthesis of heme, amino acids, and phospholipids [1,2]. Mitochondria are composed of a double membrane structure, consisting of the inner and outer mitochondrial membranes (IM and OM, respectively), which differ in function and shape. While the OM is relatively smooth, the IM is highly folded on the inside to form the crista structures that maximize the surface area, which contributes to an efficient oxidative phosphorylation [1].

It is not surprising then that mitochondrial defects or dysregulation have emerged as having key roles in aging and in the cytopathological mechanisms underlying cancer, neurodegenerative, metabolic and other diseases. Deterioration of mitochondrial function and dynamics is a hallmark of obesity and all of the illnesses associated to it [3,4]. The cellular mitochondrial network is highly dynamic and subjected to a continuous reorganization [5,6]. Mitochondrial dysfunction gives rise to changes in cristae morphology [7]. For example, nutrient deprivation induces an increase in mitochondrial elongation showing an increased number of cristae, which might increase the efficiency of adenosine-5´-triphosphate (ATP) synthesis and in this way protect mitochondria from autophagosomal degradation, augmenting cellular survival during starvation [8,9].

Dietary glucose impacts physiological and molecular processes in *C*. *elegans*, which makes it a good model for understanding hyperglycemia and obesity. It has been reported that high-glucose diets (HGD) lead to lipid accumulation, alters membrane fluidity, impacts lifespan, progeny, and increases cellular ROS levels and protein glycosylation [10,11,12,13,14,15,16]. Furthermore, the participation of diverse key transcriptional regulators of carbohydrate and lipid metabolism such as SKN-1/NRF2, HIF-1/HIF1α, SBP-1/ SREBP, CRH-1/CREB, CEP-1/p53, and DAF-16/FOXO, has been previously described [17].

In the current study, we aimed to determine the effects that HGD have on mitochondrial morphology and function. Herein, we provide direct evidence that HGD leads to swelling of mitochondria and endoplasmic reticulum in germ and muscle cells, and an increase in mitochondrial mass. Furthermore, HGD affects mitochondrial respiration without altering adenine nucleotide levels. Additionally, HGD decreases ceramide synthases and antioxidant genes mRNA accumulation, while upregulating mitophagy genes.

## Materials and methods

### Strains and culture conditions

The wild-type Bristol (N2) strain was cultured using NGM plates seeded with *E*. *coli* OP50 and raised at 18 °C, as previously described [18]. The worms were synchronized with alkaline hypochlorite solution [19], a condition in which only eggs can survive, and eggs were washed with M9 buffer solution. After synchronization, worms were seeded on a NGM plate (control condition) or in glucose-supplemented plates, and fed with *E*. *coli* OP50 until they reached L4 larval stage. Glucose (Sigma) was added to the mix of agar and salts of the NGM medium in order to obtain 20, 40, 80, or 100 mM glucose concentration, as previously reported [17].

### Observation of mitochondrial and endoplasmic reticulum ultrastructure by transmission electron microscopy

Synchronized L4 stage worms from two independent experiments were fixed in 2.5% glutaraldehyde and 4% paraformaldehyde in sodium phosphate buffer (0.1M, pH 7.4), post-fixed in 1% osmium tetraoxide, dehydrated in a graded series of ethanol and embedded in EPON (epoxy resin). Semithin sections (1 μm) were cut using an ultramicrotome (Leica EM UC6), stained with toluidine blue to select areas in light microscopy examination. Ultrathin sections of 60–90 nm were cut and collected on slot grids previously covered with formvar membrane. Sections were stained with uranyl acetate and lead citrate. The structural changes from 50 fields for each sample were recorded using a JEM-1011 transmission electron microscope (Japan). Images were taken with PhotoImpact 10 and mitochondrial diameter measurements were made with Zen 2.3 (Carl Zeiss).

### Total DNA extraction

Total DNA was purified from synchronized L4 stage worms grown at the different concentrations of glucose using Trizol (Invitrogen). DNA was further cleaned using the QIAamp DNA mini kit (Qiagen) and dissolved in nuclease-free water. DNA integrity was verified by means of agarose gel electrophoresis and was quantified by spectroscopy in a Nanodrop ND1000 equipment. All of the DNA samples were stored at -70°C.

### Mitochondrial copy number assay

Worms were synchronized as in [19] and exposed from L1 to L4 larval stage to 20, 40, 80, or 100 mM glucose, then total DNA was extracted as mentioned before. DNA integrity was assessed by gel electrophoresis. The mitochondrial DNA (mtDNA) copy number was determined by use of a quantitative PCR assay as reported [20], in which total DNA was used as a template for two qPCR reactions. One produces an amplicon of 195 bp, part of the *nduo-5* gene that codes for the mitochondrial gene NADH dehydrogenase subunit 5. The other amplifies part of the nuclear gene *flr-1* that codes for a sodium channel and leads to a fragment of 225 bp that serves as an internal concentration control. Comparison of the mitochondrial fragment amplicon (sample) to the nuclear fragment amplicon (internal concentration control) allows accurate mtDNA quantification. AccuTaq LA DNA Polymerase (Sigma-Aldrich D8045) was used for PCR reactions. The primers and conditions for the PCR reactions used are listed in Table 1. Total DNA and PCR products were quantitated by fluorescence using Quant-iT^™^ PicoGreen^™^ dsDNA Assay Kit (Invitrogen). The fluorescence values ​​of the PCR products obtained were adjusted by subtracting the fluorescence of a sample containing just buffer. mtDNA copy number was calculated as the ratio of fluorescence values of PCR products for mitochondrial fragment/nuclear fragment.

**Table 1:** Primers used for mtDNA copy number determination.

Gene	Sequence	Tm (°C)	Cycles	Product lenght
*flr-1* (nuclear)	*Fw* TCCCGTCTATTGCAGGTCTTTCCA *Rev* GACGCGCACGATATCTCGATTTTC	63	20	225 bp
*nduo-5* (mitochondrial)	*Fw* CACACCGGTGAGGTCTTTGGTTC *Rev* TGTCCTCAAGGCTACCACCTTCTTCA	63	20	195 bp

### Citrate synthase activity assay

Citrate synthase activity was determined on proteins extracted from mitochondrial fractions, using the Citrate synthase Assay Kit (CS0720-1KT, Sigma-Aldrich).

### Enzymatic activity determination of the mitochondrial respiratory chain complexes

Mitochondrial fractions from L4 larvae grown in the different concentrations of glucose were obtained as reported [21]. Mitochondrial protein content was determined using the Bradford method [22]. Mitochondrial respiratory chain complexes enzymatic activities were determined by spectrophotometric methods [23]. For the reactions, samples of 5 μg of protein were added to a quartz cell of 1 cm length and absorbances were recorded in a Spectronic Genesys 5 spectrophotometer. Absorbance was measured every 10 s for a total of 3 min.

### Adenine nucleotide pools (ATP, ADP y AMP) determination by HPLC

ATP, ADP y AMP levels were determined by HPLC. In short, aproximately 100 mg of L4 worms were collected in M9 buffer and were left at room temperature to sediment. Then, 800μl of perchloric acid 8% were added and sonicated in ice to minimize nucleotide degradation. The mix was centrifuged at 14,000 rpm for 10 min at 4°C. The supernatant was neutralized with potassium hidroxide 3 M (6% final volume), vortexed and centrifuged at 10,000 rpm for 10 min at 4°C to sediment salts and worm´s cuticle. Aproximately 720 μl of the supernatant was transferred to a new tube and pH was adjusted to 4.2 by adding 80μl of acetate buffer (5.3 ml of sodium acetate 1 M + 14.7 ml of acetic acid 1M). Nucleotide concentration was determined using an HPLC system (PerkinElmer, Waltham, MA, USA) serie 200 UV/VIS equipped with a fluorescence detector (Varian Chromatography Systems, Walnut Creek, CA, USA). Fluorescence detection was performed using the following wavelenghts: λ_ex_ = 340nm, λ_em_ = 420nm. Samples were injected in an ACE 5-μm C18 column(150 Å∼4.6 mm) (ACE HPLC Columns; Advanced Chromatography Technologies, Ltd., Aberdeen, UK). Mobile phase consisted of a solution of 1M KH_2_PO_4_, methanol 20% and Ion Pair cocktail Q6 4M (Hexyltrimethilammonium phosphate) with a flux of 1ml/min. Nucleotide concentrations was determined by the area under the curve of the peaks corresponding to each nucleotide, compared to the area obtained using a standard of the same nucleotide in a solution of known concentration.

### Malate synthase activity assay

Malate synthase activity was determined on proteins extracted from mitochondrial fractions, by reacting Acetyl CoA with the Ellman's reagent (5,5'-dithiobis-(2-nitrobenzoic acid; DTNB) following the procedure described in the technical note of Sigma-Aldrich (Sigma-Aldrich. Enzymatic assay of Malate synthase; https://www.sigmaaldrich.com/content/dam/sigma-aldrich/docs/Sigma/Enzyme_Assay/malatesynthase.pdf).

### Quantitative RT-PCR determination of *hyl-1*, *hyl-2*, *gcs-1*, *gst-4*, *pink-1*, *and dct-1* mRNA accumulation

Total RNA was purified from synchronized L4 stage worms grown at the different concentrations of glucose by using Trizol (Invitrogen) according to manufacturer recommendations and further purified using RNeasy mini kit (Qiagen). cDNA was generate with 1μg of total RNA in a 10 μl reaction using Revert Aid enzyme (Fermentas). 10ng of cDNA was used as a template. qRT-PCR was performed on a Step One Real Time PCR System (Applied Biosystems) using SYBR Green PCR Master Mix (Applied Biosystems) following the manufacturer’s instructions. Each qPCR reaction was performed using six biological replicates in triplicate each. PCR program was: 10 min at 95°C, 40 cycles of 95°C, 15 sec, Tm (Table 2) for 30 sec. and 70°C for 30 sec. Primer´s specificity was confirmed by electrophoresis in polyacrylamide gels and by melting point analysis. Y45F10D.4 gene was used as an reference gene [24]. The relative expression ratio of the mRNA relative to Y45F10D.4 gene mRNA expression was calculated as previously described [25].

**Table 2:** Primers used for determination of mRNA accumulation by qRT-PCR assays.

Gene	Sequence	Concentration (nM)	Tm (°C)
*Y45F10D*.*4*	*Fw* GCGAAAACACTCCTGCAC *Rev* TTTCGCGGGTTCTCGTAGTG	66	60
*hyl-1*	*Fw* CAT TCC ATC CTG TTC CAG AC *Rev* GCC GAT AAG AAG TGA ATA GTA G	66	60
*hyl-2*	Fw TGG GCT TAC ATT CTG TTC Rev TGC TTG GCT TTT TCA CG	66	60
*gst-4*	Fw CTC TTG CTG AGC CAA TCC GT Rev CTG GCC AAA TGG AGT CGT TG	66	60
*gcs-1*	Fw GGA ATG CCT TAC GGA GGT C Rev CGA TAG ACA TGT TTC ATC CTT C	66	60
*pink-1*	*Fw* GCATATCGAATCGCAAATGAG *Rev* CCTAAATTATAAGTGGCGGG	66	60
*dct-1*	*Fw* ACGGATGAGTCTGTGCAACC *Rev* TTCCACCCACGATTCAGGTG	66	60

### Statistical analysis

Each data ensemble was first analyzed using descriptive statistics to determine its distribution. To determine if the distribution of each data ensemble was a normal distribution, the Shapiro-Wilk test was used for small datasets (n<50) and Kolmogorov-Smirnov-Liliefors test for medium to large datasets (n<50). The number of replicas for each experiment is shown in the respective figure legend. To evaluate the differences between the analyzed groups, the ANOVA test was used if the data showed a normal distribution and comply with the homoscedasticity principle, which was evaluated with the Bartlett test. If neither of this conditions was met, the Kruskal-Wallis non-parametric test was used. To evaluate if there were statistically significant differences between the experimental treatments and the control condition, the Bonferroni test was used with a *post-hoc* ANOVA analysis. For the Kruskal-Wallis analysis, the Dunn test was used. Statistical analysis was performed with Prism GraphPad v.6 (GraphPad Software Inc., California, USA).

## Results

### Glucose affects the morphology of mitochondrial and endoplasmic reticulum in germ and muscle cells

**Fig 1:**
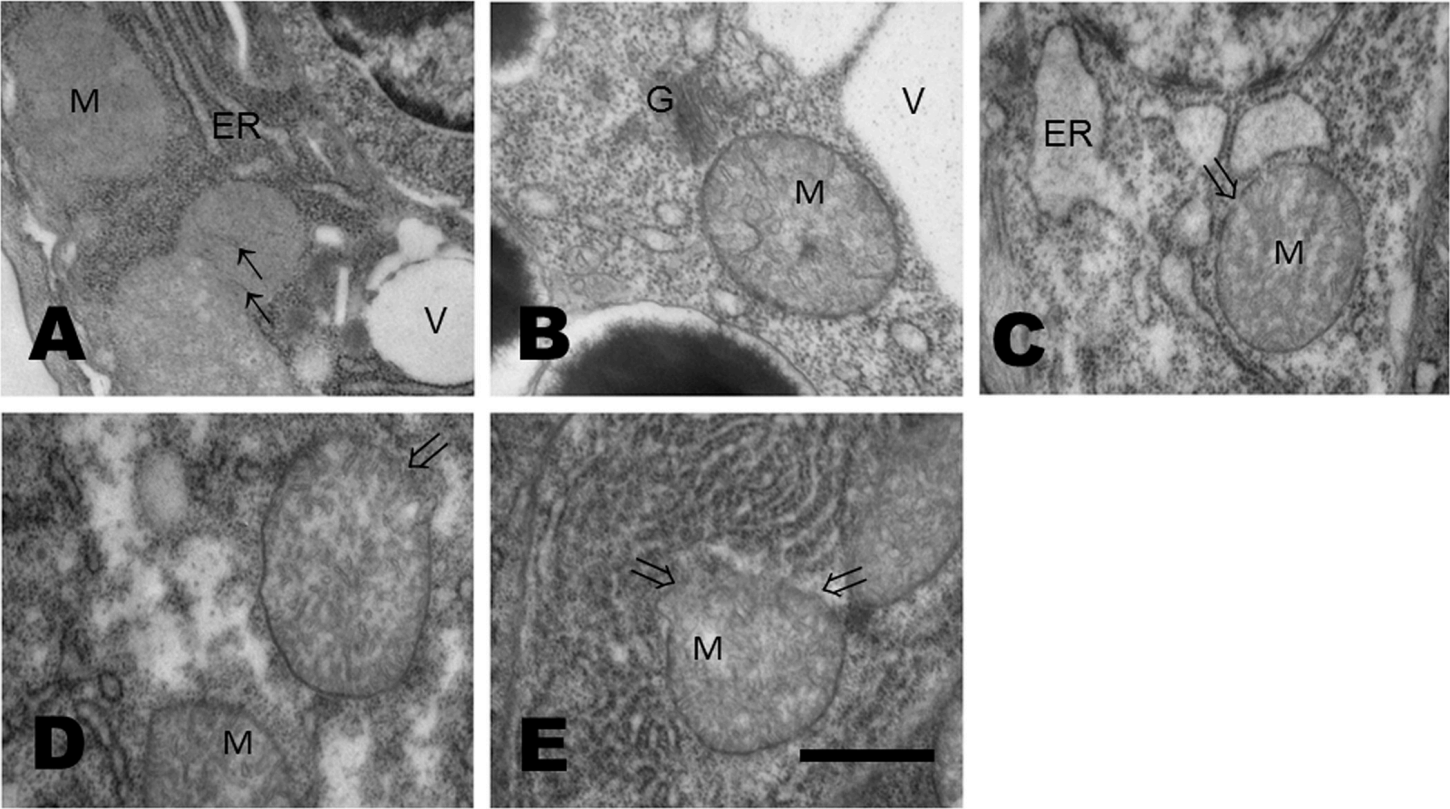
Glucose affects the morphology of mitochondria and endoplasmic reticulum of germ cells. **(A-E).** Electron micrographs of germ cells of worms that were exposed from L1 to L4 larval stages to glucose 20, 40, 80 or 100 mM that show damaged mitochondria. (A) In control worms, mitochondrial cristae membranes are fine and dim (faint) (small arrows). (B) A widening of the endoplasmic reticulum cisternae was observed at glucose 20 mM, (C) larger at 40 mM and then (D) diminished at 80 mM. Mitochondrial external membrane showed extensive rupture, and a higher amount of endoplasmic reticulum was observed in worms grown at glucose 100 mM (**E**). G, Golgi cisternae; V: vacuoles. 50000X magnification; scale bar represents 500 nm.

To determine if glucose affects the morphology of mitochondria, worms were grown in media supplemented with glucose 20, 40, 80 or 100 mM, and the morphology of germ cells and muscle cells was observed through a transmision electron microscope. Both germ and muscle cells showed a typical morphology in control worms. Germ cells have a nucleus centrally located, an electro-dense cytoplasm due to the abundance of ribosomes and glycogen, big vacuoles and mitochondria, while the external membrane and fine cristae of mitochondria are not so evident (Fig 1A). On the contrary, germ cells of glucose-treated worms showed a decrease in the electron-density of the cytoplasm and a widening of the endoplasmic reticulum cisternae (Fig 1C, 1D and 1E). Additionally, subtle changes in mitochondrial morphology were observed at glucose 40 mM, which became more prominent at 80 mM (Fig 1C and 1D). Swelling of mitochondria and its cristae, ruptures in the external membrane and enlargement of the endoplasmic reticulum were noticed at glucose 100 mM (Fig 1E).

**Fig 2:**
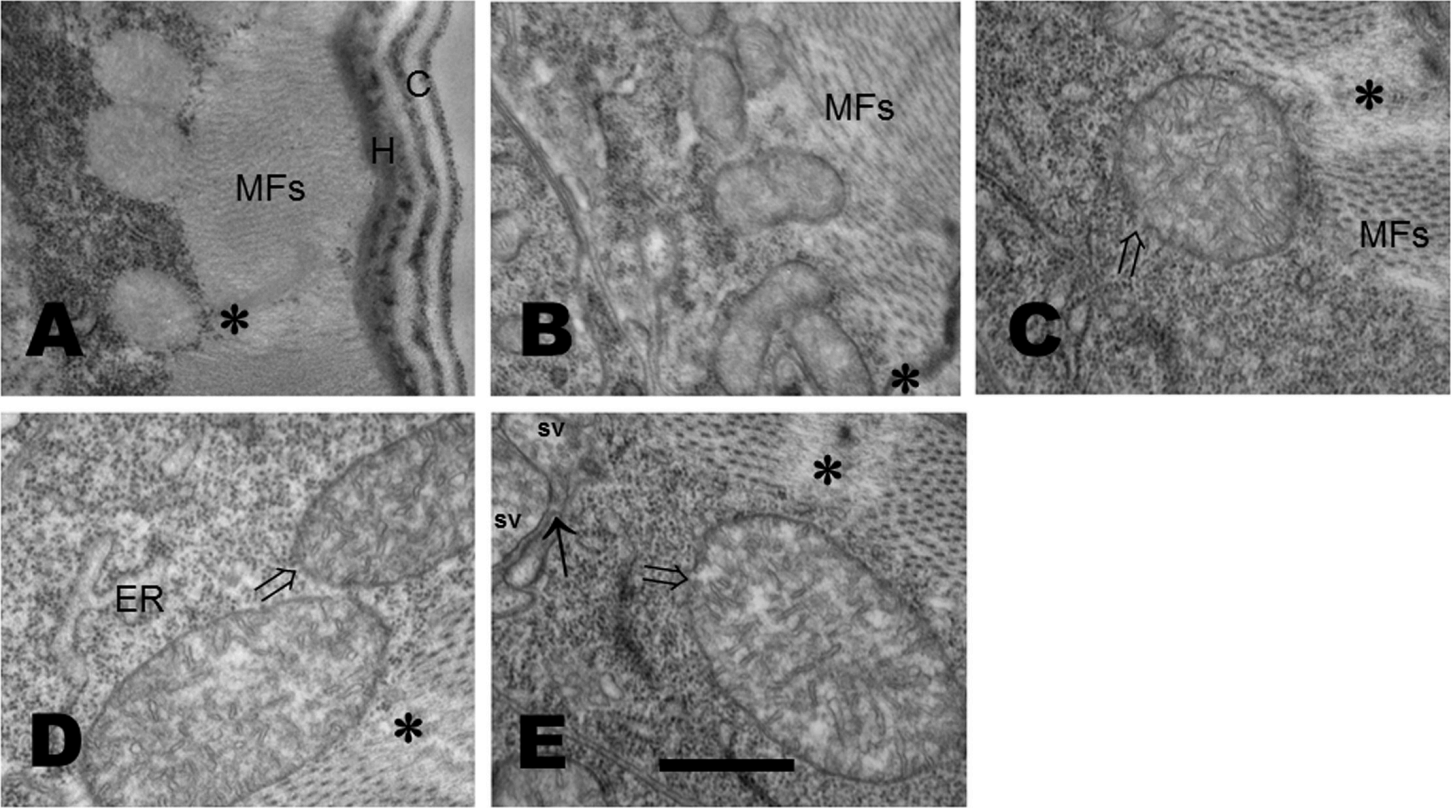
Glucose affects the morphology of mitochondria and myofibrils of muscular cells. **(A-E).** Electron micrographs of muscular cells of worms that were exposed from L1 to L4 larval stages to glucose 20, 40, 80 or 100 mM that show damaged mitochondria. (A) Mitochondria and myofibrils show a compact structure in control worms. (B) Worms treated with glucose 20 mM showed a swelling of the mitochondria, such swelling was bigger in worms exposed to 40, 80, or 100 mM; (B, C, D, and E, respectively). Mitochondrial external membrane showed zones of rupture (⇒), myofibrils are disorganized and the electrondensity of the Z-line (**) is highly diminished. C, cuticle; H, hypoderm; MFs, myofibrils; Z-line (**); sv, small vesicle; synapse (arrow). 50000X magnification; scale bar represents 500 nm.

In the case of muscle cells from control worms, mitochondria showed fine cristae and their myofibrils had a well organized and compact aspect (Fig 2A). In opposition, damaged mitochondria is observed in the muscle cells of glucose-exposed worms (Fig 2B, 2C, 2D and 2E). Additionally, a disorganization of the myofilaments and a decrease in electrodense material at the Z-line was observed in muscle cells from glucose-treated worms (Fig 2B, 2C, 2D and 2E). Together these data show that glucose produces a swelling of mitochondria, as well as an enlargement of the endoplasmic reticulum in both germ cells and muscle cells, and disorganization of myofilaments, myofibrils and the Z-line in muscle cells.

**Fig 3:**
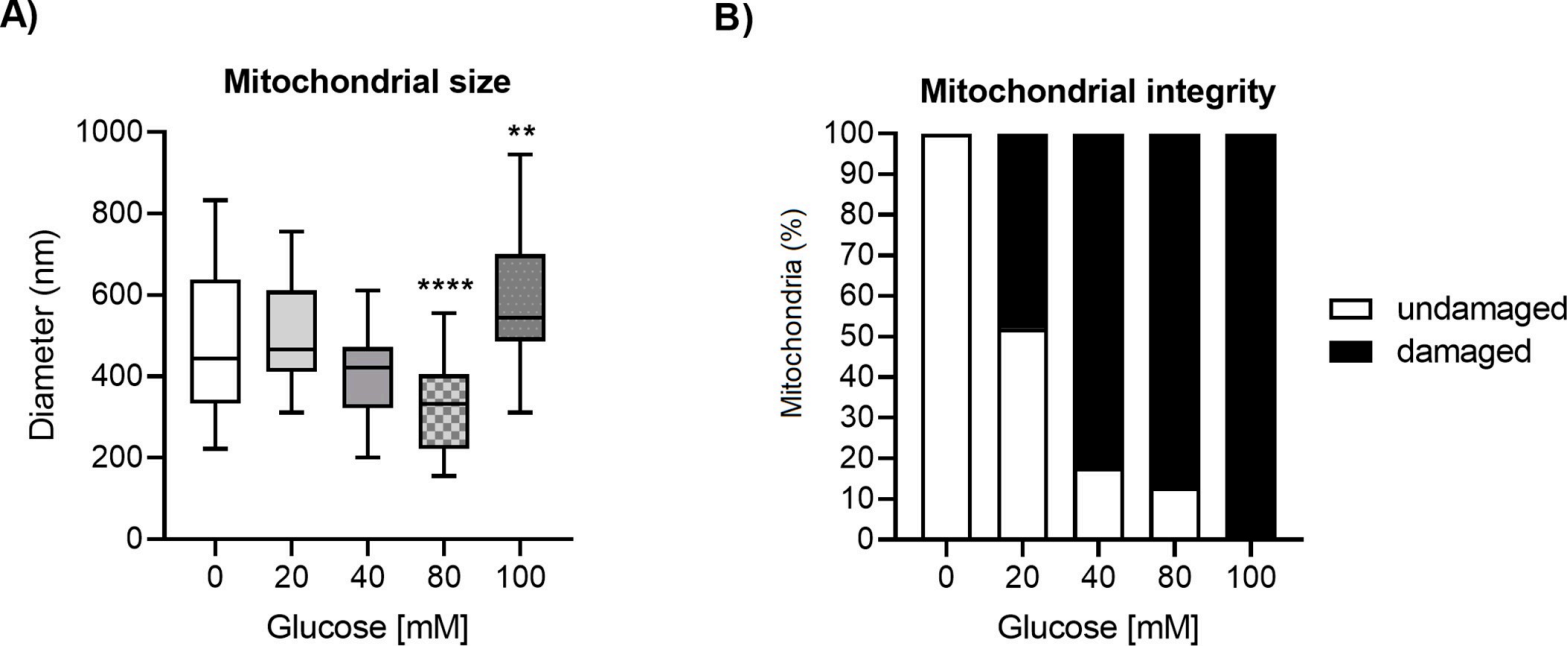
Glucose affects mitochondrial diameter and integrity. Worms were exposed from L1 to L4 larval stage to 20, 40, 80, or 100 mM glucose. (A) Boxplot illustrating mitochondrial diameter from glucose-treated worms. Measurements were made from electron microscopy images; n = 30–50 mitochondria from two biological replicates. Differences between groups were analyzed with a one-way ANOVA test. Significant differences to the control group are marked as follows: ns = not significant, *p<0.05, **p<0.01, ***p<0.001, ****p<0.0001. (B) Mitochondrial integrity in worms exposed to glucose. Measurements were done by visually inspecting mitochondria in electron microscopy images. Each mitochondria was examined and marked as damaged if swelling, cristae modification, or rupture of mitochondrial membranes was observed; n = 90–95 mitochondria from each treatment from two biological replicates.

In order to uncover the molecular causes of mitochondrial impairment and the accumulation of damaged mitochondria in worms exposed to glucose, we examined the mitochondrial diameter and determined the number of damaged mitochondria in electron microscopy images. We observed that mitochondria from glucose-treated worms displayed a slight reduction in mitochondrial diameter at glucose 80 mM (p<0.0001), and a marginal enlargement at glucose 100 mM (*P* < 0.01)(Fig 3A). We also observed that the percentage of damaged mitochondria was 48, 82, 87, or 100% for worms grown at glucose 20, 40, 80, or 100 mM, respectively, while we did not find any damaged mitochondria in worms grown in the control condition (glucose 0 mM) (Fig 3B). These findings show that exposure to glucose leads to changes in the diameter of mitochondria and accumulation of damaged mitochondria.

### Glucose affects mitochondrial mass

**Fig 4:**
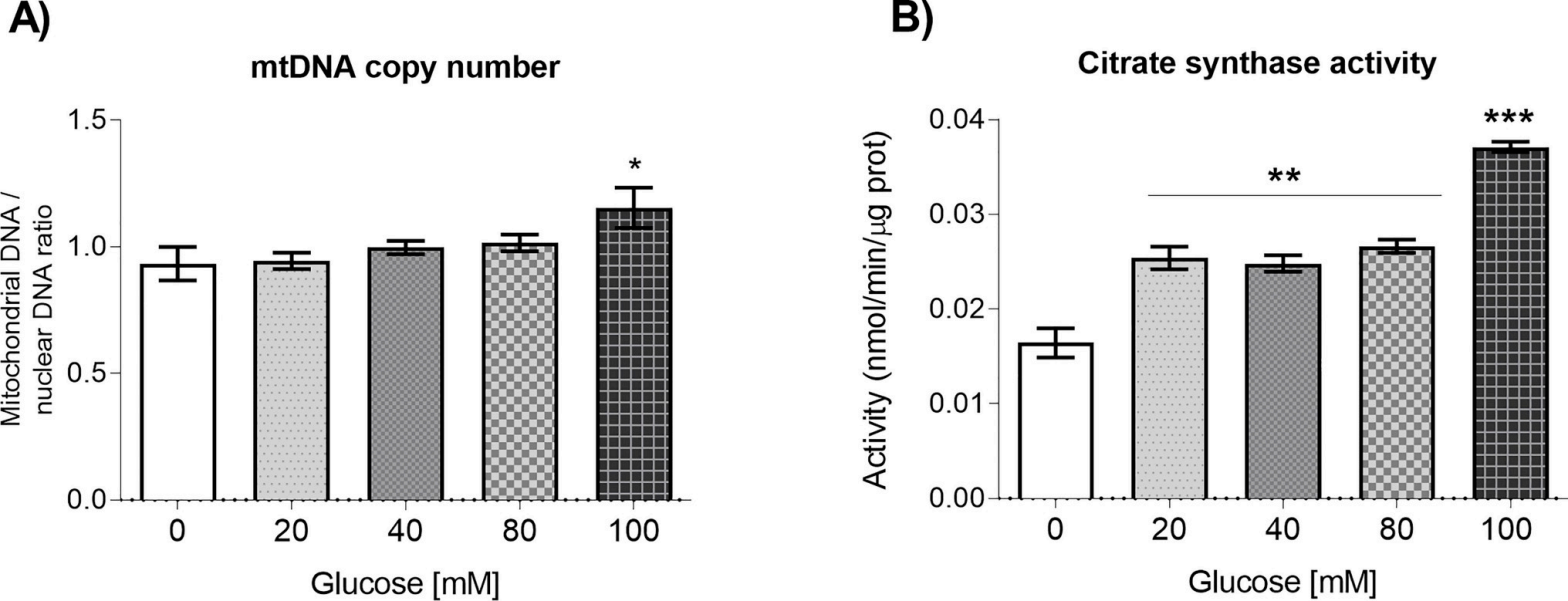
Mitochondrial DNA copy number and Citrate Synthase enzymatic activity. Synchronized worms were exposed to glucose 20, 40, 80, or 100 mM from L1 to L4 larval stage. Panels show (A) quantitative PCR analysis of mtDNA copy number (n = 6), or (B) Citrate Synthase (CS) enzymatic activity (n = 12). Values are expressed as mean ± SEM. Data was analyzed with the one-way ANOVA test. Significant differences with respect to the control group are marked as follows: *P < 0.05, **P < 0.01, ***P < 0.001.

As it is well established that the number of mitochondria is regulated during differentiation and in response to environmental conditions in *C*. *elegans* [26], we aimed to determine if glucose was able to alter the mtDNA copy number. For this, the mtDNA and nDNA contents of worms grown in the absence (control) or presence of glucose were determined by qPCR [20]. The ratio of mtDNA to nDNA was computed; the ratio in control worms was 0.9. Worms grown in media supplemented with glucose 20, 40, or 80 mM showed a ratio between 0.9 and 1.0, but worms grown in glucose 100 mM presented a ratio of 1.15, indicating a mild increase in mitochondrial mass at this glucose concentration (*P* < 0.05, Fig 4A). To further investigate if glucose affects mitochondrial content, we performed enzymatic measurements of citrate synthase (CS). CS is a key enzyme of the Krebs cycle and has been used as an accurate marker of mitochondrial mass in tissue [27]. Experiments revealed that CS enzymatic activity was increased at glucose 20, 40 or 80 mM (*P* < 0.01; Fig 4B), showing a further increase at glucose 100 mM (*P* < 0.001, Fig 4B). These results indicate that glucose-fed worms show an increase in mitochondrial content to respond to the high-glucose conditions.

### Glucose affects mitochondrial respiration without altering adenine nucleotide levels

**Fig 5:**
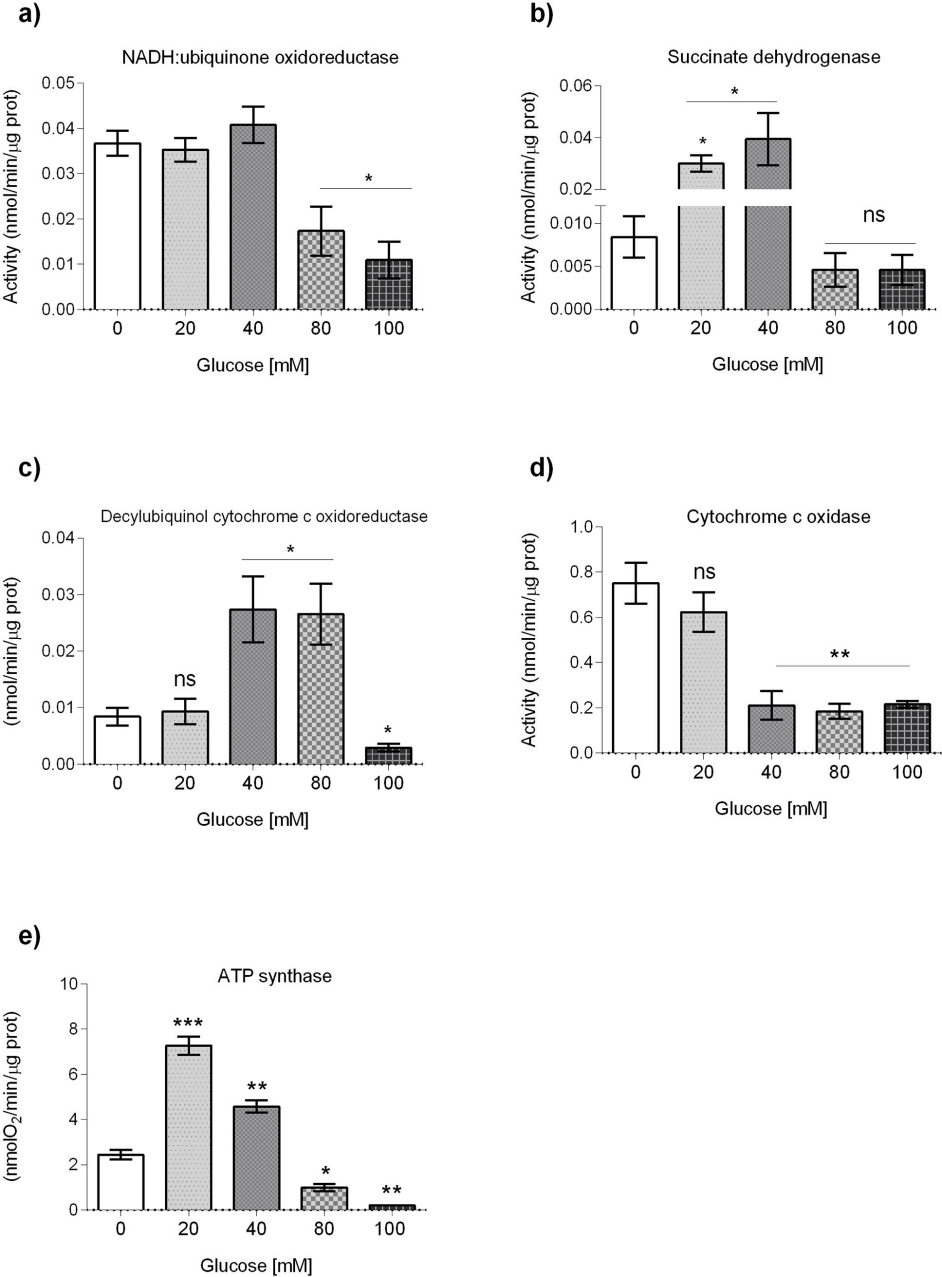
Effects of glucose on mitochondrial function. Effects of glucose on mitochondrial respiratory chain (MRC) enzymatic activities: (A) rotenone-sensitive NADH-decylubiquinol oxidoreductase (CI); (B) succinate dehydrogenase (CII); (C) antimycin A-sensitive decylubiquinol cytochrome c oxidoreductase (CIII); (D) cytochrome c oxidase (CIV), and (E) ATP synthase (CV). MRC enzymatic activities were determined spectrophotometrically with isolated mitochondria of worms that were exposed from L1 to L4 larval stages to glucose 20, 40, 80 or 100 mM. Data are presented from seven independent biological replicates and shown as median ± interquartile range (IQR) (*P < 0.05, **P < 0.01, ***P < 0.001; Kruskal-Wallis and Dunn tests for multiple comparisons).

It has been reported that mutants in mitochondrial enzymes, known as Mit mutants, show an increase in the synthesis of mtDNA as a compensatory mechanisms to the defects in the ETC [28]. A similar observation has been found in knock-down worms of ATP synthase subunit c [29]. In order to ascertain if HGDs affect mitochondrial function, the activities of the individual complexes of the mitochondrial respiratory chain (MRC) were determined in worms exposed to glucose. We showed that 80 and 100 mM glucose reduced by 50% complex I (rotenone-sensitive NADH ubiquinone oxidoreductase) activity, while no significant changes were observed at glucose 20 or 40 mM (*P* < 0.05, Fig 5A). We found that complex II (succinate dehydrogenase) activity of glucose-treated worms was augmented 3x or 5x in worms grown in 20 or 40 mM glucose, respectively (*P* < 0.05, Fig 5B), while no changes were observed at glucose 80 or 100 mM (Fig 5B). We showed that activity of complex III (antimycin A-sensitive decylubiquinol cytochrome c oxidoreductase) was no changed at glucose 20 mM, but was significantly increased at glucose 40 and 80 mM, and reduced at glucose 100 mM (*P* < 0.05, Fig 5C). We determined that complex IV (cytochrome c oxidase) activity was decreased 3x in worms exposed to glucose 40, 80 and 100 mM, while no change was observed at glucose 20 mM (*P* < 0.01, Fig 5D). Finally, complex V (ATP synthase) activity showed a mixed behavior as it was increased at glucose 20 and 40 mM (*P* < 0.001 and *P* < 0.01, respectively), while it was decreased at glucose 80 and 100 mM (*P* <0.05 and *P* < 0.01, respectively)(Fig 5E).

**Fig 6:**
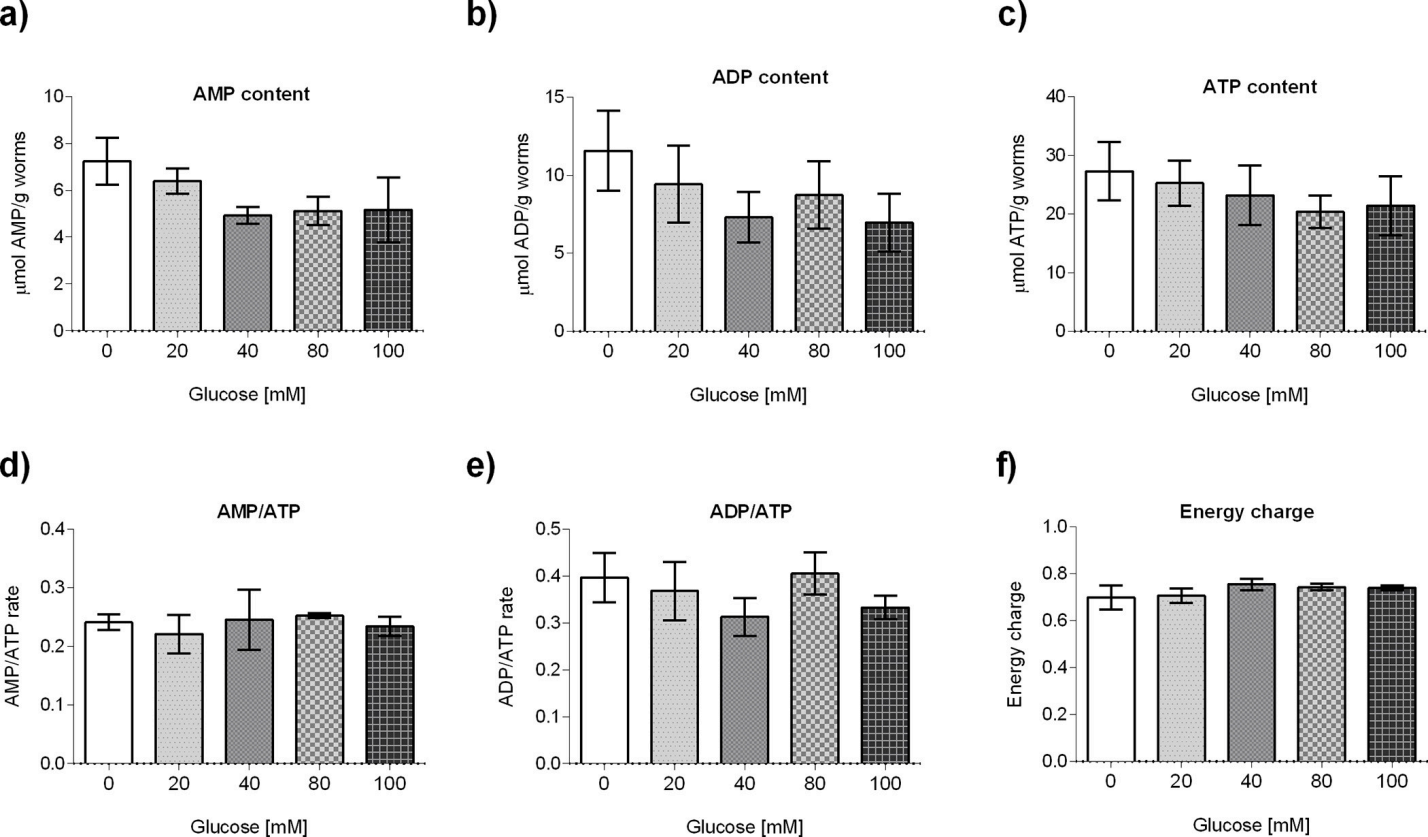
Glucose did not affect adenine nucleotide pools. Adenine nucleotide level quantification in glucose-treated worms (20, 40, 80 or 100 mM) or control using an HPLC-based assay: (A) AMP, (B) ADP, (C) ATP, used to calculate the (D) AMP/ATP quotient, (E) ADP/ATP quotient, and (F) energetic charge. Values were normalized to total weight of worms used. Data are presented from six independent biological replicates and shown as median ± interquartile range (IQR). No statistically significant differences were observed with respect to the control group. Data were analyzed by Kruskal-Wallis and Dunn tests for multiple comparisons.

To determine the effects of glucose in animal physiology, we quantified AMP, ADP and ATP levels. Notably, we did not find any changes in AMP, ADP or ATP levels or the AMP/ATP, ADP/ATP quotients, nor in the energetic charge of the cells (Fig 6A–6F). These data suggest that although glucose affects mitochondrial respiration activities, a response mechanism compensates for the changing levels of ATP production caused by exposing worms to different glucose concentrations. Together, these data suggest that glucose affects the activities of the individual components of the MRC in an intricate manner. Although glucose affects mitochondrial respiration activities, a response mechanism probably compensates for the changing levels of ATP production caused by exposing worms to different glucose concentrations.

### Glucose decreases malate synthase enzymatic activity

**Fig 7:**
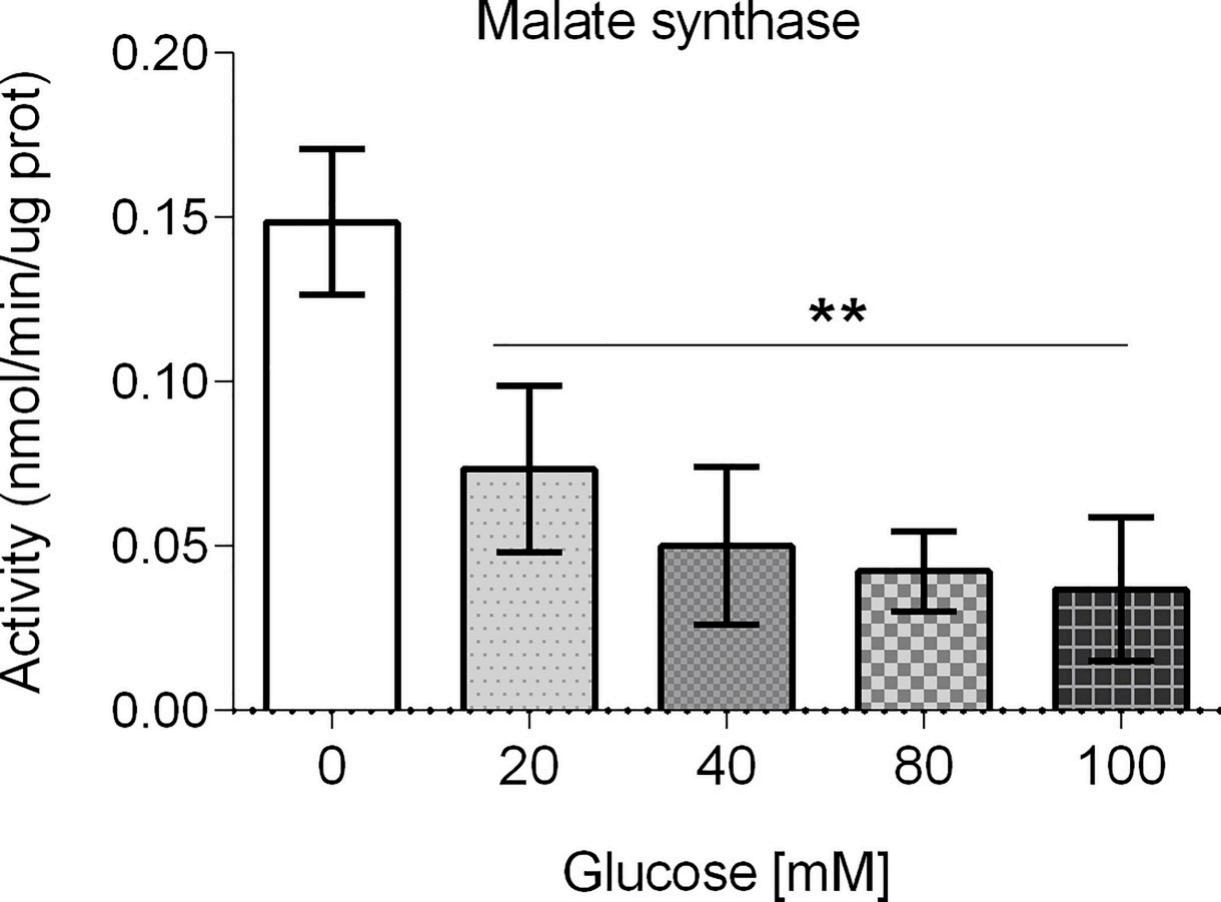
Malate synthase enzymatic activities of worms exposed to glucose. Enzymatic activities were determined in crude extracts from worms that were exposed from L1 to L4 larval stages to glucose 20, 40, 80 or 100 mM. Data is presented from 12 independent biological replicates. Error bars represent SEM (**P < 0.01, ***P < 0.001; one-way Anova and Bonferroni test to evaluate multiple comparisons).

As we did not find dramatic changes in adenine nucleotide levels in glucose-treated animals, we aimed to determine the enzymatic activity of malate synthase, involved in an alternative energy producing pathway. One single protein contains the isocitrate lyase and malate synthase enzymatic activities that are part of the glyoxylate shunt, that converts isocitrate and acetyl-CoA to succinate, malate and CoA using glyoxylate as an intermediate [30]. As can be seen in Fig 7, the malate synthase enzyme activity was decreased in all of the glucose concentrations tested (*P* < 0.01). This result could be interpreted as that other energy producing pathways different from the glyoxylate shunt are functioning in glucose-treated animals.

### Glucose decreases ceramide synthases *hyl-1* and *hyl-2* mRNA accumulation

**Fig 8:**
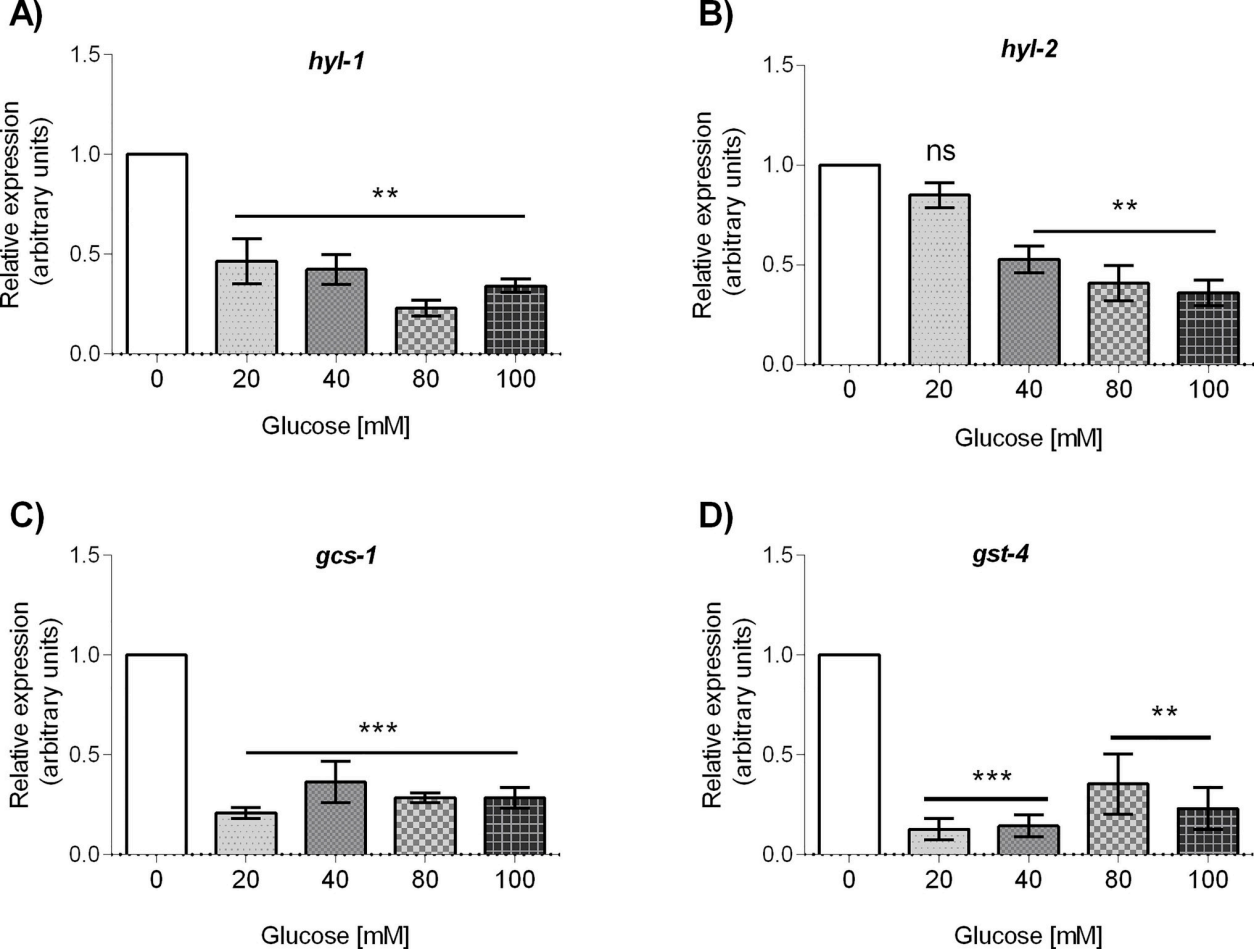
mRNA abundance of ceramide synthase enzymes and glutathione metabolism-related proteins of worms grown at different concentrations of glucose. Worms were exposed from L1 to L4 larval stage to 20, 40, 80 or 100 mM glucose. Panels show quantitative RT-PCR analysis of: (A) *hyl-1*, (B) *hyl-2*, (C) *gcs-1*, and (D) *gst-4* mRNA level in wild-type worms grown at the specified glucose concentration. Relative expression was analyzed with the Kruskal-Wallis test. Values expressed as median ± IQR (n = 6). Significant differences with respect to control group are marked as follows: ns = not significant, *P<0.05, **P<0.01, ***P<0.001.

As mitochondrial dysfunction could lead to the accumulation of ceramides [31], we aimed to determine the accumulation of the mRNAs of *hyl-1* and *hyl-2* genes, that code for ceramide synthases in glucose-fed worms. It has been described that the products encoded by *hyl-1* and *hyl-2* have proapoptotic and pro-survival effects, respectively [32]. HYL-1 function is required for synthesis of ceramides and sphingolipids containing very long acyl-chains (≥C24), while HYL-2 is required for synthesis of ceramides and sphingolipids containing shorter acyl-chains (≤C22). We found that *hyl-1* mRNA accumulation decreases about 60% in all the concentrations of glucose tested (*P* < 0.01, Fig 8A), while *hyl-2* mRNA accumulation decreases at glucose 40, 80 or 100 mM only (*P* < 0.01, Fig 8B). These results show that, contrary to our expectation, *hyl-1* and *hyl-2* mRNA levels are decreased in glucose-fed worms.

### Glucose decreases *gcs-1* and *gst-4* mRNA accumulation

The expression of the genes gamma-glutamylcysteine synthetase heavy chain (*gcs-1*) and glutathione-S-transferase 4 (*gst-4*) is regulated by the transcription factor SKN-1 under conditions of environmental stress [33,34]. In a previous report we have shown that the mRNA level of *skn-1* was downregulated by glucose [17], so we aimed to determine the effect of glucose on the mRNA accumulation of these genes. We observed that the mRNA levels of *gcs-1* (P< 0.001, Fig 8C) and *gst-4* (P < 0.01, Fig 8D) were decreased at all the glucose concentrations tested. These results suggest that the downregulation of *skn-1* that our group previously reported in glucose-fed worms [17], probably leads to the decrease in the mRNA levels of *gcs-1* and *gst-4* in glucose-fed worms.

### Glucose alters the mRNA accumulation of two genes involved in mitophagy: *pink-1* and *dct-1*

**Fig 9:**
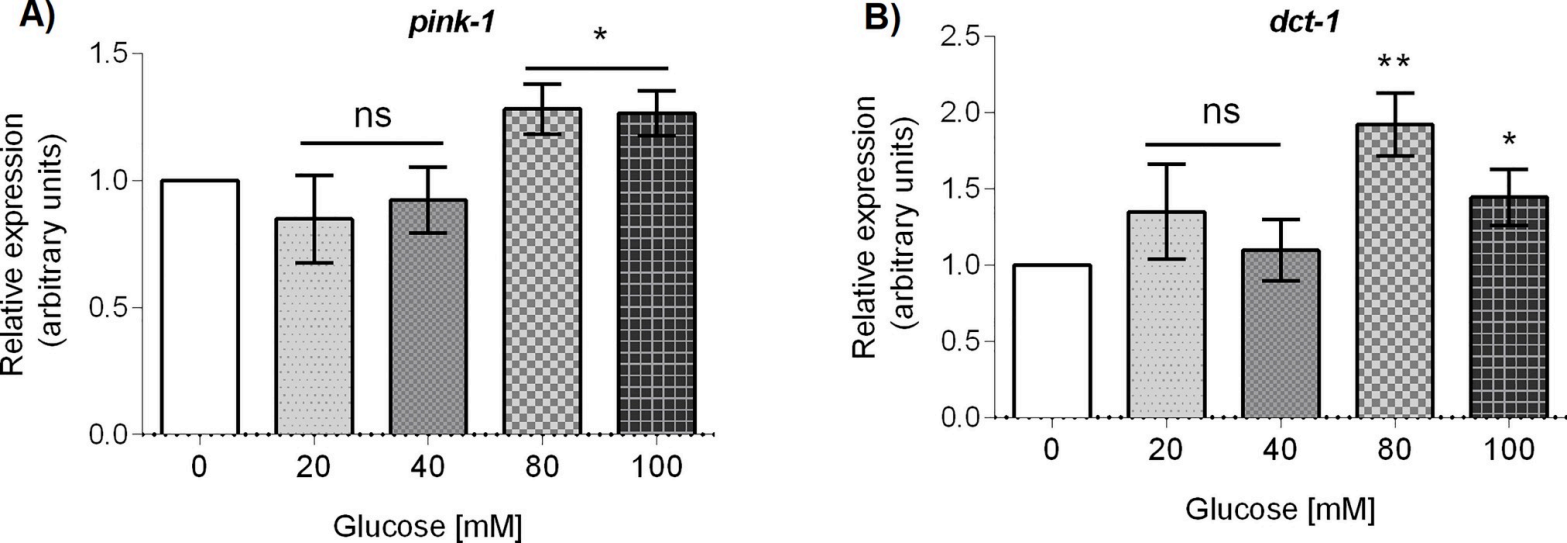
mRNA abundance of the mitophagy genes *pink-1* and *dct-1* of glucose-treated worms. Worms were exposed from L1 to L4 larval stage to 20, 40, 80, or 100 mM glucose. Panels show quantitative RT-PCR analysis of (A) *pink-1* and (B) *dct-1* mRNA levels in worms grown at the specified glucose concentration. Relative expression was analyzed with the Kruskal-Wallis test. Values expressed as median ± IQR (n = 6). Significant differences to the control group are marked as follows: ns = not significant, *p<0.05, **p<0.01.

As mitophagy is important for clearance of damaged mitochondria in order to maintain mitochondrial homeostasis. As *pink-1* (PTEN-induced kinase-1) and *dct-1* (DAF-16/FOXO-controlled germline-tumor affecting-1) are two key mitophagy genes [35], we determined if the mRNA abundance of these genes was affected by glucose. We observed that the mRNA accumulation of *pink-1* and *dct-1* was upregulated at glucose 80 and 100 mM (*P < 0.05, Fig 9A and 9B). These results suggest that in the conditions when we found an increased proportion of damaged mitochondria in glucose-treated worms, there is an upregulation of mitophagy genes as a putative response to mitochondrial dysfunction.

## Discussion

Metabolic changes produced by different nutrimental situations are at the onset of pathological mechanisms in several diseases, including cancer, neurodegenerative diseases and diabetes, highlighting the relevance of understanding how mitochondria responds to the exposure to different nutrients. Furthermore, glucose has a wide array of effects on mitochondrial function, but the regulatory mechanisms implicated are poorly understood. Here, we addressed the question of how high-glucose diets impact *C*. *elegans* mitochondrial function.

First, we observed that mitochondria from muscle cells and germ cells from larvae grown from L1 to L4 stages in increasing concentrations of glucose was conspicuously different from that of control worms. A swelling of mitochondria from worms fed with glucose was apparent. Moreover, rupture of the external membrane was observed in both muscle cells and germ cells from worms fed glucose 100 mM. Also, in worms fed with glucose, an enlargement of the endoplasmic reticulum was evident. Additionally, a disorganization of the myofilaments and the line Z was observed in muscle cells from glucose-treated worms. Similar alterations in mitochondrial ultrastructure have been reported in the gastrocnemius muscle of rats fed a high-sugar diet (68% carbohydrate), showing a swollen apperance and damaged cristae [36]. Altered mitochondria was also observed in rat embryos from a diabetic pregnancy and in embryos cultured in high concentrations of D-glucose, but not in L-glucose [37].

Alterations in mitochondria morphology have also been observed in worms carrying mutations that affect mitochondrial fission or fusion [38], and in mutants in the electron transport complex V ATP synthase [39]. Additionally, it was found that all of the mutants that showed a disrupted mitochondrial and ATP synthesis function showed an abnormal mitochondrial morphology [40]. Moreover, knockdown of genes that code for most of the mitochondrial proteins, also lead to fragmented or elongated mitochondria [41]. Damaged mitochondria has been also observed in rat embryos from a diabetic pregnancy [37] and in the muscle of rats fed a high-sugar diet [36], as well as fragmented hepatic mitochondria in high-fat diet-fed mice [42], showing that this phenomenon also occurs in mammals.

Mitochondrial dysfunction gives rise to an unusual cristae morphology [7]. Subjecting the cells with the mitochondrial DNA (mtDNA) replication inhibitor 2´, 3´-dideoxycytidine (ddC), the cells showed a concentrically remodeled cristae [43], which recently was also visualized and confirmed by superresolution imaging of mitochondria cristae using stimulated emission depletion (STED) microscopy [44]. Mitochondrial volume homeostasis is important for function and is regulated by ion fluxes; levels of potassium (K^+^) are important for mitochondrial osmotic balance, while levels of calcium (Ca^2+^) for bioenergetics [45,46]. Recently, leucine zipper/EF-hand-containing transmembrane protein 1 (LETM1) has been identified as the gene responsible for Wolf-Hirschhorn syndrome; these proteins are highly conserved and act as a mitochondrial K^+^/H^+^ exchanger and as a mitochondrial Ca^2+^/H^+^ antiporter [47], In the case of *C*. *elegans* and human cells, the expression of Letm1 gene and mitochondrial volume is inversely correlated, and a reduction in the levels of the LETM1 protein lead to swollen mitochondria showing less electro-dense matrices [48], In this context, it would be very interesting to determine if LETM1 is involved in the rupture of mitochondria seen under high-glucose conditions or other mechanisms explain the swelling of mitochondria.

Dysfunctional mitochondria has been associated with an increase in mitochondrial mass. So, we determined the mitochondrial DNA to nuclear DNA ratio and found only a slight increase at 100 mM glucose-treated worms. Recently, it has been reported that mtDNA is a poor marker of mitochondrial mass, while citrate synthase activity is strongly associated to mitochondrial content [49]. For this reason, we measured CS enzymatic activity and found an increase in CS activity in all of the glucose concentrations tested, suggestive of an increase in mitochondrial content. This result is similar to reports in which worms with defects in mitochondrial respiration [28,29] or wild-type worms fed with *E*. *coli* strain HT115 instead of OP50 [50], elicit a compensatory mechanism to produce more mitochondria.

As we observed that worms fed a high-glucose diet showed damaged mitochondria, it is possible that glucose-treated worms have dysfunctional mitochondria. We determined the enzymatic activities of the respiratory complexes of glucose-treated worms. We found a decreased activity of complex I at glucose 80 and 100 mM, while no change was observed at other glucose concentrations. Perplexingly, the activity of complex II was augmented at glucose 20 and 40 mM, while no change was evident at greater glucose concentrations. Activity of complex III was increased at glucose 40 and 80 mM, but was diminished at glucose 100 mM, while activity of complex IV was decreased at glucose 40, 80 and 100 mM. This data indicates that glucose has complex effects on individual enzymatic activities of the ETC that depends on the concentration of glucose used and the individual component of the ETC considered. Such a complex dynamics showing the interdependence of individual components of the ETC have been reported previously [7,51,52]. For example, a knockdown in ATP synthase subunit C led to a decrease in complex I enzymatic activity [29]. Also, a long-lived *clk-1* mutant showed deficiencies in complex I-dependent metabolism due to a different ability of complex I and II to use quinone pools [53]. Moreover, long-lived *isp-1* mutants have a decreased complex I activity explained by an altered allosteric interaction between ETC components [54]. Additionally, a knockdown in complex IV shortens the lifespan of *C*. *elegans* and diminishes complex I enzymatic activity [55]. In this regard, it is interesting to note that in a previous report we observed a diminished longevity in glucose-treated worms [17], and here we found a decrease in both complex I and complex IV enzymatic activities. These results provide evidence on the intertwined relationships between the different complexes of the ETC and the effects that glucose has on ETC enzymatic activities.

It has been reported that HGD-fed worms showed a reduced respiratory activity [56,57]. We observed that complex V (ATP synthase) enzymatic activity was augmented in glucose 20 and 40 mM, while it was diminished at 80 and 100 mM. Surprisingly, glucose-exposed worms did not show altered ATP, ADP or AMP levels. One possible explanation for this is the existence of a compensatory mechanism to low levels of ATP synthesis. It has been reported that worms possess a glyoxalate pathway that can bypass mitochondrial dysfunction and provide an alternative energy source [58]. In our case, the enzymatic activity of isocitrate lyase/malate synthase, an enzyme with a dual function in the glyoxalate shunt, diminishes in a concentration-dependent manner. Another possibility is that glucose-treated worms show a decreased energy expenditure, so ATP levels could be maintained. For example, mutations in *clk-1* that decrease mitochondrial function showed normal of increased ATP levels that result from diminished energy utilization [59]. Interestingly, studies of worms with disrupted mitochondria genes that extend longevity does not show changes in ATP levels [29,59,60,61].

Ceramides are known to participate in important cellular process like apoptosis, stress response and the mitochondrial unfolded response [62,63,64,65,66]. Ceramides are produced in the endoplasmic reticulum (ER) and function within the ER and mitochondria [62]. As changes in ceramide concentration have been associted with diseases involving mitochondrial dysfunction [63,64,67], we determined the mRNA accumulation of *hyl-1* and *hyl-2*, genes involved in ceramide synthesis. It has been reported that *hyl-1* is necessary for the synthesis of C24 to C26 ceramide and sphingomyelin compounds, while *hyl-2* is required for the synthesis of C20 to C22 compounds [32]. A mutation in *hyl-1* increases oxygen deprivation survival, while a mutation in *hyl-2* results in oxygen deprivation sensitivity [15,32]. The sensitivity to oxygen deprivation of a *hyl-2* mutant is worsened if the worms are fed a glucose diet, showing an altered expression of genes involved in innate immunity, cuticle function and phase I and II detoxification system [68]. We observed that both *hyl-1* and *hyl-2* mRNAs were downregulated in glucose-treated worms. This downregulation could be part of a response mechanism to decrease sphingolipid synthesis to diminish the toxicity of high-glucose diets.

Recently, we reported that high-glucose diets lead to a mild activation of some antioxidant pathways in *C*. *elegans*, concomitant with a downregulation of *skn-1* mRNA accumulation [17]. Here we aimed to extend our query on the participation of other mitohormetic genes involved in antioxidant pathways. We found that the mRNAs of *gcs-1* and *gst-4*, that code for gamma glutamyl synthetase and glutathione S-transferase 4, respectively, involved in glutathione and detoxification metabolism, were downregulated in a glucose-supplemented diet. The correlation observed between the decreased mRNA levels of *skn-1* and two of their transcriptional targets in glucose-treated worms surely warrants a further in-depth study. A similar downregulation of *gcs-1* and *gst-4* mRNA has been reported in glucose-fed worms, which was shown to be a consequence of the inhibition of the SKN-1-mediated immune response to the infection with *Salmonella typhimurium* [69]. Along the same lines, the SKN-1-mediated increase of expression of *gcs-1* and *gst-4* in response to Paraquat was suppressed in the presence of glucose [70]. It is interesting to note that the knockdown of some genes of the detoxification system increased the anoxia survival rate of glucose-treated worms or *hyl-2* mutants [68]. Furthermore, it has been reported that the protective effect seen by the addition of 5-aminoimidazole-4-carboxamide ribonucleoside (AICAR) under high-glucose conditions was independent of antioxidant defense pathways [71]. It is thought that the detoxification system can modulate the stress response through secondary mechanisms, such as to promote excretion of toxic metabolites, so knockdown or downregulation of some detoxification genes may be beneficial in some circunstances [68].

Finally, mitophagy involves the targeting of damaged mitochondria to lysosomes for degradation and recycling of their components, particularly under conditions of environmental stress [72]. As mitophagy has been reported to be impaired under high-glucose treatments [73], we aimed to study the mRNA accumulation of two genes involved in this process. Mitochondrial phosphatase and tensin (PTEN)-induced kinase 1 (*pink-1*) and the ortholog of human BNIP3 (BCL2 interacting protein 3) (*dct-1*) are two key genes that code for proteins that regulate mitophagy in *C*. *elegans* and other organisms [74]. In damaged mitochondria, PINK-1 is accumulated in the outer membrane, where it recruits PARKIN to target the damaged mitochondria for degradation by the lysosome [75]. Furthermore, DCT-1 acts downstream of PINK-1 which is ubiquitinated upon mitophagy-inducing conditions to help in the removal of damaged mitochondria [74]. We observed that the mRNA level of both genes slightly increased at glucose 80 or 100 mM, exactly in the same conditions in which we observed an increased number of damaged mitochondria. The upregulation of *pink-1* and *dct-1* could be part of a mitohormetic response involving mitophagy to adjust mitochondrial population under conditions of HGD.

## Conclusions

In summary, our data clearly shows that HGD dramatically alter mitochondrial morphology, mass and function, without affecting adenine nucleotide pools. Moreover, our results suggest that perhaps the glyoxylate cycle does not represent an alternative energetic source as the malate synthase enzymatic activity was decreased in glucose-fed worms. We also observed a downregulation of genes involved in ceramide biosynthesis and genes involved in glutathione metabolism, maybe as these downregulation is beneficial in glucose-treated worms. Additionally, the upregulation of genes involved in mitophagy was observed as a response to the exposure to glucose. It will be a worthwhile future task to elucidate the molecular mechanisms involved in the alterations in morphology and function of the mitochondria induced by HGD in *C*. *elegans*.
